# Hydroethanolic Stem Bark Extract of *Burkea africana* Attenuates Vincristine-Induced Peripheral Neuropathy in Rats

**DOI:** 10.1155/2020/7232579

**Published:** 2020-02-12

**Authors:** Yakubu Jibira, Eric Boakye-Gyasi, Wonder Kofi Mensah Abotsi, Isaac Kingsley Amponsah, Donatus Wewura Adongo, Eric Woode

**Affiliations:** ^1^Department of Pharmacology, Faculty of Pharmacy and Pharmaceutical Sciences, Kwame Nkrumah University of Science and Technology (KNUST), Kumasi, Ghana; ^2^Department of Pharmacognosy, Faculty of Pharmacy and Pharmaceutical Sciences, Kwame Nkrumah University of Science and Technology (KNUST), Kumasi, Ghana; ^3^Department of Pharmacology, University of Health and Allied Sciences, Ho, Volta Region, Ghana

## Abstract

*Context*. The stem bark of the savanna tree *Burkea africana* (Hook) (family: Leguminosae) is used in the Ghanaian traditional medicine for the management of various pain-related diseases.

**Objective:**

This study seeks to investigate the possible antiallodynic and antihyperalgesic effects of the hydroethanolic stem bark extract of *B. africana* in a vincristine-induced peripheral neuropathy model in rats. *Materials and Methods*. 0.1 mg kg^−1^ vincristine was administered intraperitoneally for 5 days followed by 2 days break and continued for another 5 days to establish peripheral neuropathy in Sprague Dawley rats. Effects of *Burkea africana* (Hook) (family: Leguminosae) is used in the Ghanaian traditional medicine for the management of various pain-related diseases. *p.o.*) and pregabalin (10–100 mg kg^−1^, *i.p.*) were assessed on tactile, intermediate, mechanical, cold, and hot allodynia as well as in the Randall–Sellito test. Moreover, the levels of total proteins, malondialdehyde (MDA), reduced glutathione (GSH), superoxide dismutase (SOD), and catalase (CAT) in sciatic nerve tissue homogenates were assayed.

**Results:**

BAE (50–1000 mg kg^−1^*p.o*.) showed significant antiallodynic and antihyperalgesic effects similar to pregabalin by increasing paw withdrawal latency and paw withdrawal threshold in all the behavioral tests used. Also, the extract decreased the levels of MDA (a lipid peroxidation product) as well as MPO and caused a significant increase in endogenous antioxidants (GSH) and antioxidant enzymes (SOD and CAT) in tissue homogenates of treated rats.

**Conclusions:**

Results from this study indicate that the hydroethanolic stem bark extract of *B. africana* exhibits antiallodynic and antihyperalgesic effects in vincristine-induced peripheral neuropathy in rats.*B. africana* in a vincristine-induced peripheral neuropathy model in rats.

## 1. Introduction

Neuropathic pain is normally caused by a lesion or pathological alteration of the somatosensory system, including central neurons and A*β*, A*δ*, and C neuronal fibers in the periphery [1]. Due to the ageing global population and the fact that most cancer patients are surviving after chemotherapy, the prevalence of neuropathic pain is expected to increase [2]. Mechanisms implicated in the pathogenesis of neuropathic pain include differences between excitatory and inhibitory somatosensory signaling, changes in ion channels, and inconsistency in the way that pain messages are modulated in the CNS [1, 3].

Since neuropathic pain may be partially or completely unresponsive to primary analgesic treatments [4], medical therapies for neuropathic pain tend to involve drugs whose primary indication is not analgesia such as antiepileptic drugs, anti-arrhythmic agents, and antidepressants [5, 6]. Vincristine-induced peripheral neuropathy in rats is a common model in pain research, which resembles human peripheral neuropathy [4]. Traditionally, plants have historically proven their value as a source of lead supplements with therapeutic potential and currently represent an important reservoir for the discovery and development of novel drugs. The pharmaceutical and research industries now rely mainly on folk libraries of plant product as their pool for drug discovery [7, 8]. In Africa especially northern Ghana, various parts including the stem bark of *B. africana* are widely used traditionally to treat pain [9]; however, there is little scientific prove for its efficacy. This current study therefore sought to investigate the effects of *B. africana* stem bark extract in the management of neuropathic pain in experimental peripheral neuropathy in rats.

## 2. Materials and Methods

### 2.1. Chemicals and Reagents

Vincristine sulphate (Celon Laboratories PVT LTD, Maharashtra, India), pregabalin (Pfizer Inc, New York, USA), adrenaline (Coax Bioremedies Private limited, Haryana, India), 5,5′-dithiobis (2-nitrobenzoic acid) (DTNB), diaminobenzidine (Hach Company, Loveland, Colorado, USA), sodium azide (Atom Scientific, Hyde, Manchester, UK), Thiobarbituric acid, and bovine serum albumin (Sigma Aldrich, St Louis. MO, USA).

### 2.2. Animals

Sprague Dawley rats (200 ± 5 g) used in this study were bought from Center for Plant medicine Research, Mampong-Akuapem, Eastern region, Ghana. The animals were acclimatized in groups of five in stainless steel cages (34 × 47 × 18 cm^3^) at the Department of Pharmacology *vivarium* and fed with a low-fat rodent chow (purchased from Agricare Limited, Tanoso, Kumasi). All animals were maintained in a 12 h day and night cycles and given water *ad libitum.* All experimental protocols employed in the study conform to the standards set by the Department of Pharmacology Ethics Committee and the Guide for the Care and Use of Laboratory Animals, 8th Edition.

### 2.3. Collection and Extraction of Plant Material

Matured stem bark of *Burkea africana* (Hook) were collected from Tamale (9° 59′ 29.6797″ N; 2° 30′ 51.5059″ W) in the Northern region of Ghana in April, 2017. It was authenticated by Dr. George Henry Sam of the Department of Herbal Medicine, Faculty of Pharmacy and Pharmaceutical Sciences, KNUST, Kumasi, Ghana. A voucher specimen (KNUST/HM1/2017/SB005) was kept at the faculty's herbarium. The fresh plant material was sorted to remove all foreign materials and air-dried at room temperature for five days. The dried bark was pulverized into a coarse powder using a hammer mill (Christy and Norris, Chelmsford, England). About 2 kg of the powdered bark was extracted with 70% v/v ethanol in a Soxhlet extractor. The extract obtained was concentrated using a rotary evaporator (Rotavapor R-215, BÜCHI Labortechnik AG, Flawil, Switzerland) and further dried (at 35°C) into a solid mass (Yield = 10.85% w/w) in an electric oven (Leader Engineering, Widnes Cheshire, UK). The dried extract obtained was kept in a refrigerator until when needed and denoted as BAE or extract throughout the study.

### 2.4. Phytochemical Screening

Phytochemical analysis of the extract was carried out using standard methods as described by Prashant Tiwari et al. [10].

### 2.5. Acute Oral Toxicity Test

Twenty-four ICR male mice (20–25 g) were divided into four groups (*n* = 6). Prior to the investigation, the animals were deprived of food for 3 h. Group 1 was the control, and the mice received only the vehicle; Groups 2, 3, and 4 were treated with doses of 50, 500, and 5000 mg kg^−1^. After the drug administration, the animals were monitored continuously for every 30 min over a 24 h period to observe morphological and behavioral changes and neurological and autonomic responses if any and also observed for any death in the course of the study period. The experimental protocol and procedure used were in accordance with the OECD guidelines for testing acute oral toxicity of the chemicals [11].

### 2.6. Experimental Design

To induce peripheral neuropathy in rats, 0.1 mg kg^−1^ vincristine sulphate was administered intraperitoneally for 5 days followed by 2 days break and continued for the next 5 days as described by Woode et al. [12] and Flatters and Bennett [13]. Nociceptive responses were recorded before and after the last dose of vincristine administration to ascertain the establishment of peripheral neuropathy in the animals.

Rats were then randomly put into seven groups (*n* = 10). Group I served as the vincristine control group and received normal saline (10 mL kg^−1^, *i.p.*). Groups II, III, and IV rats received BAE 50, 500, and 1000 mg kg^−1^, *p.o.*, respectively. Groups V, VI, and VII rats were treated with pregabalin 10, 30, and 100 mg kg^−1^, *i.p.*, respectively. Thirty minutes after *i.p*. injections and 1 h after oral administrations, response of the rats to various behavioral tests were assessed at 0.5, 1, 2, 3, 4, and 5 h. Behavioral tests used included von Frey filaments (5, 9, and 14 g), cold water (4 ± 0.5°C), hot water (55 ± 0.5°C), and the Randall–Sellito test.

The percentage maximal possible effects (% MPE) were calculated using the following formula:

(1) $$\begin{array}{c} \%\text{MPE}=\left(\frac{L_{2}-L_{1}}{L_{0}-L_{1}}\right)\times 100, \end{array}$$

where *L*_1_ = (before drug) withdrawal time or force, *L*_2_ = (after drug) withdrawal time or force, and *L*_0_ represent the cutoff time or force.

In the von Frey filaments test, withdrawal responses from both hind paws were recorded and then expressed as a percentage response. Thus, if out of 10 von Frey filament applications, 6 withdrawals are noted, then the percentage response for that filament is 60 [13–15].

### 2.7. Behavioral Assessment

#### 2.7.1. Tactile Allodynia and Intermediate and Mechanical Hyperalgesia

In this assessment, the animals were restrained and the tactile allodynia was evaluated using the von Frey filaments (IITC Life Science Inc. Model 2888, Woodland Hills, CA, USA) with a bending force of 5 g. Normal rats do not respond to 5 g force stimuli, so withdrawal of the foot from 5 g force by the experimental rats depicts tactile allodynia in neuropathy. Von Frey filaments with bending force of 9 and 14 g was used in determining the intermediate and mechanical hyperalgesia, respectively. Rats with no neuronal impairment will withdraw from the 15 g bending force 5–10% of the time and the responses of the 9 g force are considered as intermediate hyperalgesia. The filaments were applied to the midplantar of the hind foot 6 times, with each touch held for 5 s. The withdrawal responses to the filaments were recorded and estimated as a percentage response [12].

#### 2.7.2. Cold Allodynia

The tail withdrawal latencies in response to cold stimulation were assessed using the method described by SałatSalat et al. [16]. This was done by immersion of the rat's tail into cold water maintained at 4 ± 0.5°C. Latency to tail withdrawal was measured with a digital timer. A cutoff time of 30 s was established to avoid tail tissue damage. For each animal, two recordings were made for the tail, and the withdrawal responses were reported as the mean of the two values and percentage maximal possible effect (%MPE) calculated.

#### 2.7.3. Hot Water Hyperalgesia

Response of the animals to hot water was carried out as previously described by Back [17]. About 4 ± 1.5 cm of the terminal tail of rats was immersed in hot water maintained at 55 ± 0.5°C, and tail withdrawal latencies were recorded. For each animal, two recordings were made, and the withdrawal responses were recorded as the mean of the two values and percentage maximal possible effect (%MPE) calculated. A cutoff time of 30 s was used.

#### 2.7.4. Randall–Sellito Test

Mechanical nociception induced by vincristine was measured with an analgesimeter (MN-15776, Ugo Basile, Comerio, Vareese, Italy) as described previously by Hosea et al. [18]. The analgesimeter was used to apply pressure by means of a blunt perspex cone to the dorsal region of the left hind paw until there was a withdrawal response. The paw withdrawal thresholds (PWTs) were recorded as the pressure (grams) required to exhibit paw withdrawal. A cutoff weight of 250 g was employed to prevent any tissue damage to the limb. A change in the hyperalgesia state was calculated as a percentage of the maximum possible effect (MPE).

### 2.8. Biochemical Analysis

Rats used in the study were humanely sacrificed on day 15 of the experiment by high-dose pentobarbitone (0.1 gkg^−1^ *i.p*.). The sciatic nerve from the hind limbs and some associated tissues were isolated as described elsewhere [19] and stored at −4°C. The sciatic nerve tissues were homogenized using 0.1 M tris-HCl buffer (pH 7.4). Homogenate collected in the sample tubes were kept at 3 and 4°C and centrifuged at 2000 rpm. The supernatant and precipitate were collected into separate tubes. The supernatant was used in evaluating the total protein content and malondialdehyde (MDA), reduced glutathione (GSH), superoxide dismutase (SOD), and catalase (CAT) levels. The homogenate of the tissues attached to the sciatic nerve was centrifuged at 5000 rpm for 10 mins at 4°C and used to evaluate the myeloperoxidase (MPO) content.

#### 2.8.1. Total Protein Content

The method as described by [20] was employed in this assay. Bovine serum albumin (BSA) was used as the reference, and the spectroscopic measurement was taken at 660 nm. The pH of the assay was maintained between 10 and 10.5. The protein content in the samples was estimated from the BSA calibration graph.

#### 2.8.2. Superoxide Dismutase (SOD) Levels

SOD activity was determined as described by McCord Maccords and Fridovich [21]. An amount of 500 *μ*L homogenate was mixed with 150 *μ*L of ice-cold chloroform and 750 *μ*L ethanol (96% v/v) and vortexed for 60 s before centrifuging at 2000 rpm for 20 min to separate the supernatant from the precipitate. One millilitre of carbonate buffer (0.1 M; pH 10.2) and 0.5 mL EDTA (0.6 mM) were treated with 500 *μ*L of supernatant. Adrenaline solution (0.05 mL of 1.3 mM) was then added to initiate adrenochrome formation. The absorbance was read spectrophotometrically at 480 nm using the Synergy H1 multimode reader (BioTek Technologies, Winooski, VT, USA). The amount of SOD needed to inhibit adrenaline autoxidation was expressed as follows:

(2) $$\begin{array}{c} \%\text{ inhibition}=\left(\frac{{\text{Absorbance }}_{\text{test }}-{\text{Absorbance }}_{\text{blank}}}{{\text{Absorbance}}_{\text{test}}}\right)\times 100. \end{array}$$

SOD activity was expressed in units per mg protein, where 1 unit is the enzyme quantity needed to show 50% dismutation of the superoxide radical at 25°C calculated using the following formula:

(3) $$\begin{array}{c} \text{Units of SOD activity}/\text{mg protein}=\left(\frac{\%\text{ inhibition}}{50\times \text{wt of protein}}\right). \end{array}$$

#### 2.8.3. Catalase (CAT) Levels

CAT activity was assessed using methods described by Sinha [22] and Antwi et al. [23]. An amount of 0.4 mL H_2_O_2_ (1.18 M) and 1 mL phosphate buffer (0.01 M; pH 7.0) were added to 0.1 mL of homogenate and incubated for 5 min at 25°oC. Afterwards, 2 mL dichromate-acetic acid reagent (containing 3 parts glacial acetic acid and 1 part 5% w/v potassium dichromate) was then added to terminate the reaction. The activity exhibited by catalase was expressed as units per mg protein based on the molar extinction coefficient of H_2_O_2_ and 39.4 M^−1^ cm^−1^ at 620 nm using Synergy H1 multimode reader (BioTek Technologies, Winooski, VT, USA). One unit is the enzyme quantity needed to hydrolyse 1 mmol of H_2_O_2_/min in a neutral pH (25°C), i.e.,

(4) $$\begin{array}{c} \text{mUnit of CAT activity}/\text{mg protein}=\left(\frac{{\text{Absorbance}}_{620nm}}{39.4\times \text{wt of protein}}\right)\times 1000. \end{array}$$

#### 2.8.4. Reduced Glutathione (GSH) Levels

The concentration of GSH in the test samples was measured by the procedure described elsewhere [24]. One-hundred microlitres (100 *μ*L) of the homogenate and 2.4 mL of 0.02 M EDTA were added and vortexed for 1 min. The solution was then cooled for 10 min at 4°C. 2 mL H_2_O and 0.5 mL of 50% w/v TCA were added to the mixture and centrifuged at 3000 rpm for 5 min. Afterwards, 50 *μ*L of 10 mM DTNB solution and 2 mL of Tris buffer (0.4 M; pH 8.9) were then mixed with 1 mL of the supernatant and the reaction mixture incubated at 25°C for 5 min. A reaction mixture was repeated also for the blank. The absorbance was spectrophotometrically read at 412 nm using the synergy H1 multimode reader (BioTek Technologies, Winooski, VT, USA). GSH concentration was expressed in *μ*mol per mg protein and determined using the curve, *y*=0.0004*x* + 0.0026

#### 2.8.5. Lipid Peroxidation Product (Malondialdehyde) Levels

The amount of malondialdehyde (MDA) formation was determined as previously described by Ohkawa et al. [25]. One millilitre (1 mL) of homogenate was mixed with 3 mL of a mixture (3 mL 20% TCA containing 0.5% TBA) in a test tube. It was heated at 95°C for 30 min, cooled immediately, and then centrifuged at 5000 rpm for 10 min. Absorbance was initially read at 532 nm and then read again at 600 nm to correct for nonspecific absorbance using the Synergy H1 multimode reader (BioTek Technologies, Winooski, VT, USA). The molar extinction coefficient of MDA-TBA abduct, 155 mM^−1^ cm^−1^, was used to determine the amounts of MDA from the following equation:

(5) $$\begin{array}{c} \text{nmol MDA}/\text{mg protein}=\frac{{\text{Absorbance}}_{532\text{nm}}-{\text{Absorbance}}_{600\text{nm}}}{155\times \text{total protein}}\times 10^{6}. \end{array}$$

#### 2.8.6. Myeloperoxidase Levels

Enzyme concentration was determined spectrophotometrically by a modified 3, 3′-diaminobenzidine (DAB) colorimetric method by Klangprapan et al. [26]. The reaction was initiated by adding homogenate, 0.5 mM DAB solution and 6 mM H_2_O_2_. The reaction was terminated by adding 0.1 mM sodium azide, and the absorbance was read at 465 nm in 60 s cycle for 600 s using the Synergy H1 multimode reader (BioTek Technologies, Winooski, VT, USA). The MPO specific activity was expressed in units per mg protein, where 1 unit increases the absorbance by 0.001 per 60 s.

### 2.9. Statistics

All statistical analyses were performed with GraphPad Prism v. 6.01 (GraphPad Software, San Diego, USA). Time-course curves were analysed by two-way repeated measures analysis of variance (RM ANOVA) with treatment (between-subjects) and time (within-subjects) as factors, and the mean treatment effects at each time point were compared by Dunnett's post hoc test. Subsequently, the area under the curves (AUCs) was calculated to determine the overall treatment effect and total antinociceptive score. The difference in total antinociceptive score was determined using one-way ANOVA with Turkey's post hoc test using treatment data as the between-subject factor for data which were distributed normally. Differences were considered statistically significant at *P* value <0.05.

## 3. Results

### 3.1. Phytochemical Screening

The qualitative phytochemical tests revealed the presence of alkaloids, flavonoids, saponins, tannins, reducing sugars, phytosterols, and terpenoids (Table 1).

### 3.2. Acute Toxicity

All the animals survived throughout the 24 h study period, and from the observations, the mice did not show any signs of a change in behavior and neurological and autonomic toxicity. The oral LD_50_ of BAE was thus estimated to be above 5000 mg kg^−1^.

### 3.3. Effects of Hydroethanolic Stem Bark Extract of *B. africana* on Tactile Allodynia

**Figure 1:**
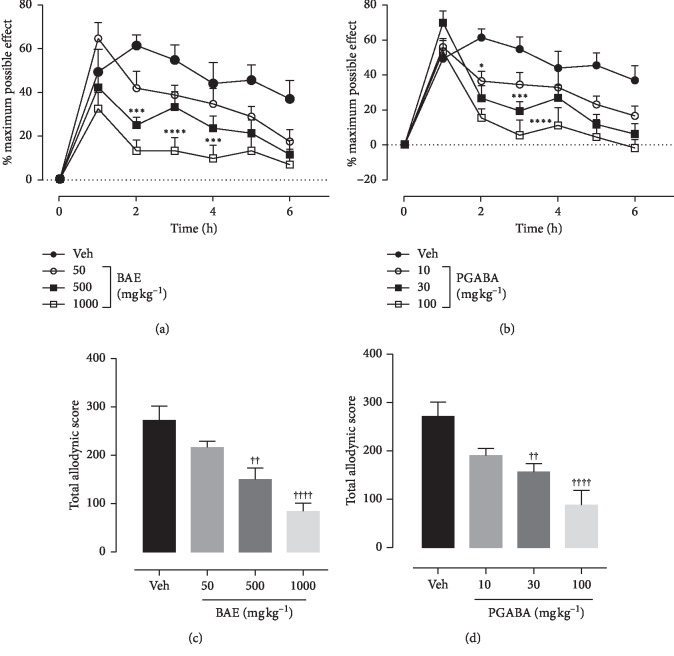
Effect of BAE (50–1000 mg kg^−1^ *p.o*.) and pregabalin (PGABA) (10–100 mg kg^−1^ *p.o.*) on the vincristine-induced tactile allodynia in rats (von Frey 5 g). (a, b) The time-course effect; (c, d) the bar graphs of the AUCs. ^*∗∗∗∗*^*P* < 0.0001; ^*∗∗*^*P* < 0.01 compared with the vehicle-treated group (2-way RM ANOVA followed by Dunnett's post hoc test). ^++++^*P* < 0.0001; ^++^*P* < 0.01 compared with the vehicle-treated group (ordinary 1-way ANOVA followed by Turkey's post hoc test). Each data presented are mean ± SEM (*n* = 10).

Ten days administration of 0.1 mg kg^−1^ vincristine resulted in the development of tactile allodynia as depicted in the vincristine control group. Treatment with BAE or pregabalin (PGABA) caused a significant decrease in response to the 5 g force von Frey filament compared to with the vincristine control group. ANOVA (treatment × time) revealed a significant effect of treatment on tactile allodynia of both BAE (*F*_treatment(6,252)_ = 22.31, *P* < 0.0001; *F*_time(3,252)_ = 27.64, *P* < 0.0001; *F*_interaction(18,252)_ = 1.685, *P*=0.0422) (Figure 1(a)) and PGABA (*F*_treatment(6,252)_ = 27.58, *P* < 0.0001; *F*_time(3,252)_ = 22.56, *P* < 0.0001; *F*_interaction(18,252)_ = 2.456, *P*=0.0011) (Figure 1(b)). Both BAE (*F*_(3,36)_ = 13.32_,_*P* < 0.0001) (Figure 1(c)) and PGABA (*F*_(3,36)_ = 10.59_,_*P* < 0.0001) (Figure 1(d)) treated rats showed significant decrease in total allodynic scores with their highest doses giving maximum inhibitions of 69.26% and 67.69%, respectively.

### 3.4. Effects of Hydroethanolic Stem Bark Extract of *B. Africana* on Intermediate Hyperalgesia

**Figure 2:**
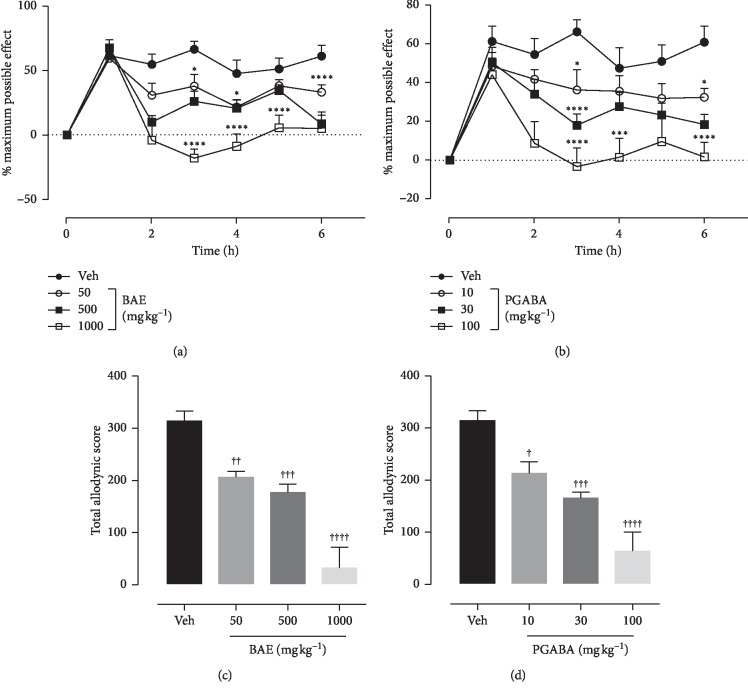
Effect of BAE (50–1000 mg kg^−1^ *p.o*.) and pregabalin (PGABA) (10–100 mg kg^−1^ *p.o.*) on the vincristine-induced intermediate hyperalgesia in rats (von Frey 9 g). (a, b) The time-course effect; (c, d) the bar graphs of the AUCs. ^*∗∗∗∗*^*P* < 0.0001; ^*∗∗∗*^*P* < 0.001; ^*∗∗*^*P* < 0.01; ^*∗*^*P* < 0.05 compared with vehicle-treated group (2-way RM ANOVA followed by Dunnett's post hoc test). ^++++^*P* < 0.0001; ^+++^*P* < 0.001; ^++^*P* < 0.01; ^+^*P* < 0.05 compared with the vehicle-treated group (ordinary 1-way ANOVA followed by Turkey's post hoc test). Each data presented are mean ± S.E.M (*n* = 10).

Effect of BAE and pregabalin (PGABA) on intermediate hyperalgesia was evaluated using the von Frey filament of 9 g bending force. Vehicle control animals exhibited a decrease in paw withdrawal latency compared with BAE- and PGABA-treated animals. ANOVA (treatment × time) revealed a significant effect of treatment on intermediate hyperalgesia for both BAE (*F*_treatment(6,245)_ = 26.76, *P* < 0.0001; *F*_time(3,245)_ = 39.74, *P* < 0.0001; *F*_interaction(18,245)_ = 4.081, *P* < 0.0001) (Figure 2(a)) and PGABA (*F*_treatment(6,252)_ = 15.32, *P* < 0.0001; *F*_time(3,252)_ = 32.43, *P* < 0.0001; F_interaction(18,252)_ = 2.456, *P*=0.0163) (Figure 2(b)). BAE and pregabalin produced significant antihyperalgesic effect (*F*_(3,36)_ = 14.99, *P* < 0.0001 (Figure 2(c)) and *F*_(3,36)_ = 19.80, *P* < 0.0001 (Figure 2(d)) at all doses tested with maximum possible antihyperalgesic effect of 90.26% and 80.13%, respectively.

### 3.5. Effects of Hydroethanolic Stem Bark Extract of *B. africana* on Mechanical Hyperalgesia

**Figure 3:**
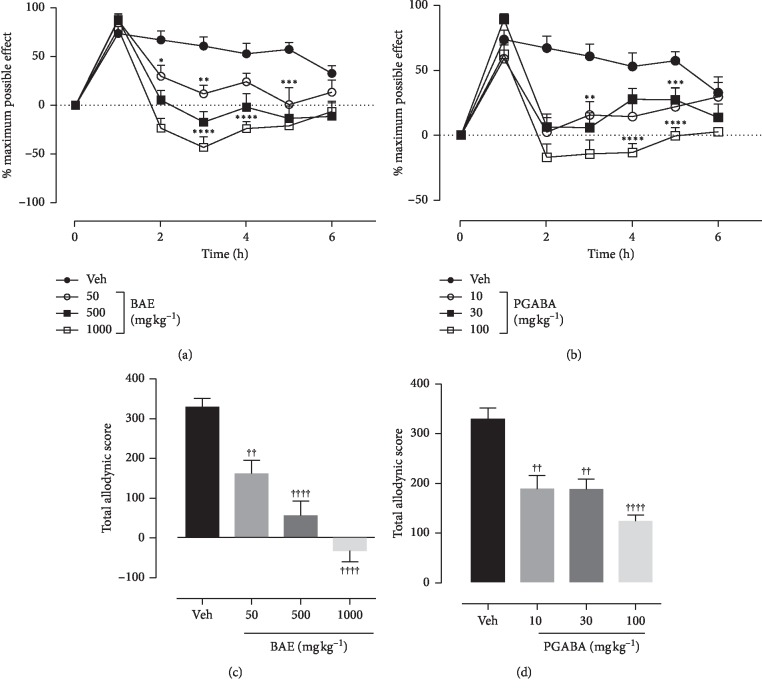
Effect of BAE (50–1000 mg kg^−1^ *p.o*.) and PGABA (10–100 mg kg^−1^ *p.o.*) on the vincristine-induced static hyperalgesia in rats (von Frey 14 g). (a, b) The time-course effect; (c, d) the bar graphs of the AUCs. ^*∗∗∗∗*^*P* < 0.0001; ^*∗∗∗*^*P* < 0.001; ^*∗∗*^*P* < 0.01; ^*∗*^*P* < 0.05 compared to vehicle treated group (2- way RM ANOVA followed by Dunnett's post hoc test). ^++++^*P* < 0.0001; ^++^*P* < 0.01 compared with the vehicle-treated group (ordinary 1-way ANOVA followed by Turkey's post hoc test). Each data presented are mean ± S.E.M (*n* = 10).

ANOVA (treatment × time) revealed a significant effect of treatment on mechanical hyperalgesia for both BAE (*F*_treatment(6,229)_ = 36.43, *P* < 0.0001; *F*_time(3,229)_ = 45.06, *P* < 0.0001; *F*_interaction(18,229)_= 4.485, *P* < 0.0001) (Figure 3(a)) and PGABA (*F*_treatment(6, 210)_ = 19.68, *P* < 0.0001; *F*_time(3, 210)_ = 31.90, *P* < 0.0001; *F*_interaction (18,210)_ = 3.047, *P* < 0.0001) (Figure 3(b)). BAE (50–1000 mg kg^−1^) treatment resulted in a significant (*F*_(3,33)_ = 23.11, *P* < 0.0001) and dose-dependent decrease in mechanical hyperalgesia with the highest dose of 1000 mg kg^−1^ producing a complete reversal of mechanical hyperalgesia (Figure 3(c)). Similarly, pregabalin (10–100 mg kg^−1^) also caused a dose-dependent and significant (*F*_(3,33)_ = 13.23, *P* < 0.0001) decrease in mechanical hyperalgesia with a maximum possible effect of 62.78% (Figure 3(d)).

### 3.6. Effects of Hydroethanolic Stem Bark Extract of *B. africana* on Cold Allodynia

**Figure 4:**
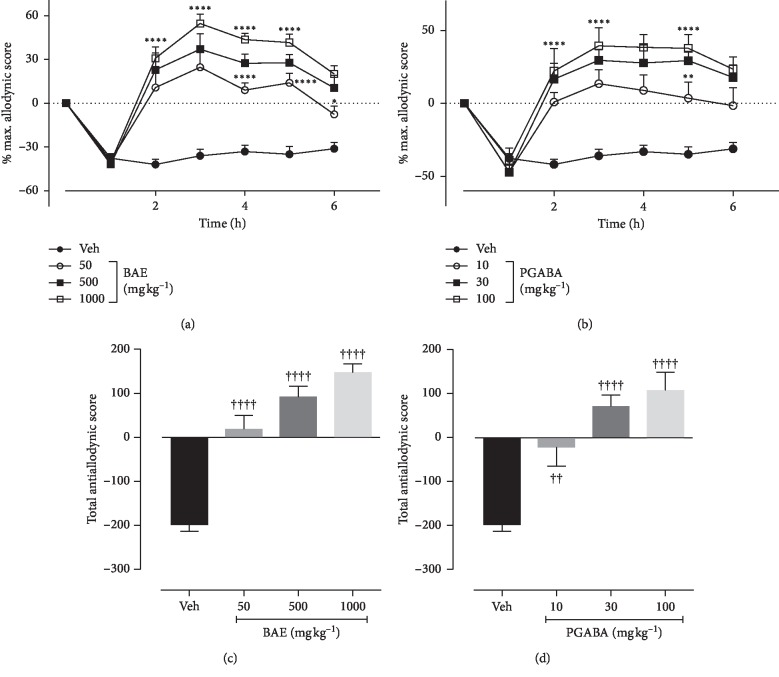
Effect of BAE (50–1000 mg kg^−1^ *p.o*.) and PGABA (10–100 mg kg^−1^ *p.o.*) on vincristine-induced cold allodynia in rats. Upper panel shows the time-course effect whiles the lower panel represents the bar graphs of the AUCs respectively. ^*∗∗∗∗*^*P* < 0.0001; ^*∗∗*^*P* < 0.01 compared with the vehicle-treated group (2-way RM ANOVA followed by Dunnett's post hoc test). ^++++^*P* < 0.0001; ^++^*P* < 0.01 compared with the vehicle-treated group (ordinary 1-way ANOVA followed by Turkey's post hoc test). Each data presented are mean ± S.E.M (*n* = 10).

Baseline response to cold water (4°C) was taken at the end of the vincristine treatment. ANOVA (treatment × time) revealed a significant effect of treatment on cold allodynia for both BAE (*F*_treatment(6,244)_ = 33.15, *P* < 0.0001; *F*_time(3,244)_ = 89.23, *P* < 0.0001; *F*_interaction(18,244)_ = 7.043, *P* < 0.0001) (Figure 4(a)) and PGABA (*F*_treatment(6,244)_ = 21.50, *P* < 0.0001; *F*_time(3,244)_ = 49.11, *P* < 0.0001; F_interaction(18,244)_ = 3.854, *P* < 0.0001) (Figure 4(b)). BAE (50–1000 mg kg^−1^ *p.o.*) produced a significant (*F*_(3,36)_ = 40.24, *P* < 0.0001) and dose-dependent inhibition of cold allodynia (Figure 4(c)) with all three doses used producing an increased in latency of tail withdrawal to the cold stimuli. Pregabalin (10–100 mg kg^−1^) also increased significantly the latency of tail withdrawal (*F*_(3,36)_ = 16.14, *P* < 0.0001) in a dose-dependent manner with the highest dose giving a possible anti-allodynic effect of 182.7 ± 33.23% (Figure 4(d)).

### 3.7. Effects of Hydroethanolic Stem Bark Extract of *B. africana* on Hot Allodynia

**Figure 5:**
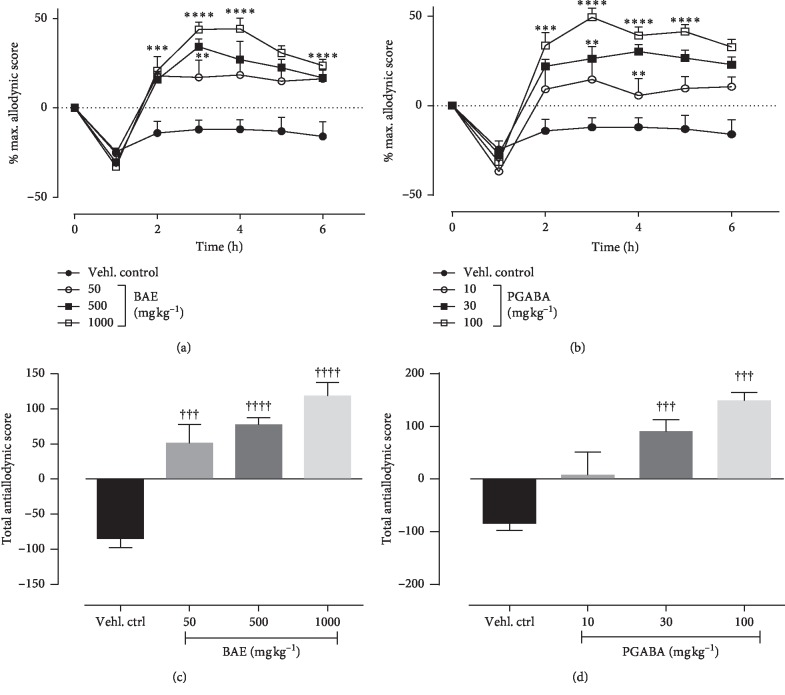
Effect of BAE (50–1000 mg kg^−1^ *p.o*.) and PGABA (10–100 mg kg^−1^ *p.o.*) on the vincristine-induced thermal-allodynia in rats. (a, b) The time-course effect; (c, d) the bar graphs of the AUCs. ^*∗∗∗∗*^*P* < 0.0001; ^*∗∗*^*P* < 0.01 compared with the vehicle-treated group (2-way RM ANOVA followed by Dunnett's post hoc test). ^++++^*P* < 0.0001; ^+++^*P* < 0.001 compared with the vehicle-treated group (ordinary 1-way ANOVA followed by Turkey's post hoc test). Each data presented are mean ± S.E.M (*n* = 10).

Treatment with BAE (50–1000 mg kg^−1^ *p.o.*) (*F*_treatment(6,112)_ = 32.34, *P* < 0.0001; *F*_time(3,112)_ = 37.75, *P* < 0.0001; *F*_interaction(18,112)_ = 3.738, *P* < 0.0001) (Figure 5(a)) and pregabalin (10–100 mg kg^−1^*p.o.*) (*F*_treatment(6,112)_ = 32.21, *P* < 0.0001; *F*_time(3,112)_ = 47.72, *P* < 0.0001; *F*_interaction(18,112)_ = 3.880,*P* < 0.0001) (Figure 5(b)) revealed a significant effect of treatment on hot allodynia. Both BAE (50–1000 mg kg^−1^ *p.o.*) (*F*_(3,36)_ = 22.58, *P* < 0.0001) and pregabalin (10–100 mg kg^−1^) (*F*_(3,36)_ = 14.25, *P* < 0.0001) significantly and dose-dependently inhibited hot allodynia (Figures 5(c) and 5(d)) with maximum percentage 224.1 ± 18.79% and 332.1 ± 59.39%, respectively.

Effects of hydroethanolic stem bark extract of B. africana on mechanical hyperalgesia (Randall–Sellito test).

**Figure 6:**
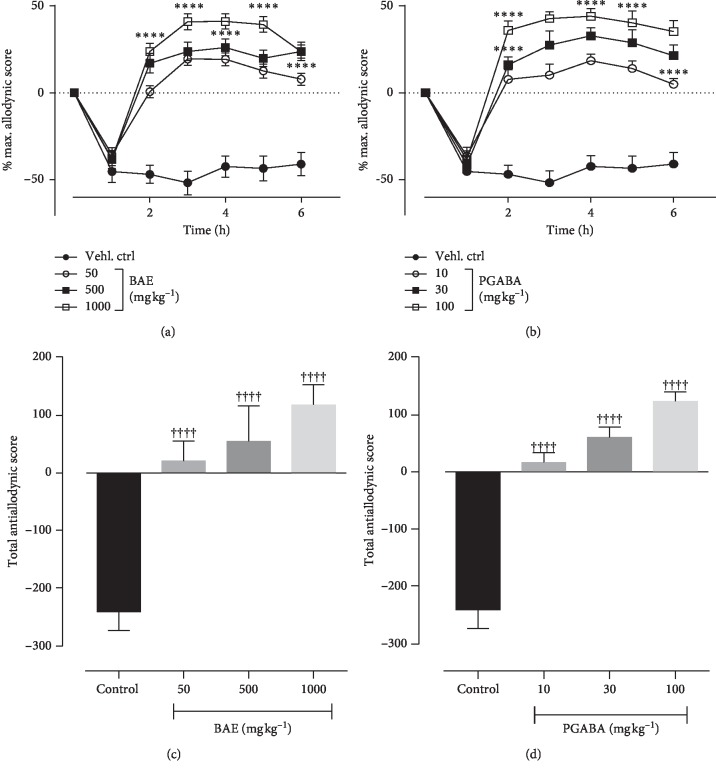
Effect of BAE (50–1000 mg kg^−1^ *p.o*.) and PGABA (10–100 mg kg^−1^ *p.o.*) on the vincristine-induced hypernociception (Randall–Sellito) in rats. (a, b) The time-course effect; (c, d) the bar graphs of the AUCs. ^*∗∗∗∗*^*P* < 0.0001 compared with the vehicle-treated group (2-way RM ANOVA followed by Dunnett's post hoc test). ^++++^*P* < 0.0001 compared with the vehicle-treated group (ordinary 1-way ANOVA followed by Turkey's post hoc test). Each data presented are mean ± S.E.M (*n* = 10).

ANOVA (treatment × time) revealed a significant effect of treatment on mechanical hyperalgesia for both BAE (*F*_treatment(6,246)_ = 57.24, *P* < 0.0001; F_time(3,246)_ = 213.10, *P* < 0.0001; *F*_interaction(18,246)_ = 13.41, *P* < 0.0001) (Figure 6(a)) and PGABA (*F*_treatment(6,237)_ = 50.41, *P* < 0.0001; *F*_time(3,237)_ = 183.80, *P* < 0.0001; *F*_interaction(18,237)_ = 11.83, *P* < 0.0001) (Figure 6(b)). BAE (50–1000 mg kg^−1^ *p.o.*) dose-dependently attenuated the vincristine-induced mechanical hyperalgesia (*F*_(3,36)_ = 59.63, *P* < 0.0001) (Figure 6(c)) with the highest dose given a percentage maximum possible effect of 142.1 ± 5.76%. Similarly, pregabalin also significantly (*F*_(3,36)_ = 52.18, *P* < 0.0001) and dose-dependently inhibited the mechanical hyperalgesia with a maximum mechanical antihyperalgesic effect of 157.9 ± 8.58% (Figure 5(d)).

### 3.8. Effects of Hydroethanolic Stem Bark Extract of *B. africana* on Sciatic Nerve Homogenate Biochemistry

#### 3.8.1. Total Protein Content

Sciatic nerve homogenate from the vincristine control rats showed a significantly high level of total protein compared to naïve and treated rats (Table 2). Treatment with BAE 500 mg kg^−1^ significantly decreased (*F*_(3,12)_ = 11.48, *P*=0.0008) the total protein content to 3.13 ± 0.18 mg g^−1^ of tissue compared to the vincristine control group with a total protein content of 4.37 ± 0.20 mg g^−1^ of tissue. In much the same way, pregabalin treatment (10 mg kg^−1^) was also able to decrease significantly (*F*_(4,15)_ = 4.531, *P*=0.0134) the total protein content in the rats (Table 2).

#### 3.8.2. Superoxide Dismutase (SOD) Level

SOD levels in BAE-treated rats were increased significantly (*F*_(4,20)_ = 81.91, *P* < 0.0001) compared with the vincristine control group. Acute treatment with 1000 mg kg^−1^ of the extract increased SOD the levels up to 0.43 ± 0.0 SOD unit mg^−1^ proteins (Table 2). Similarly, pregabalin-treated animals also showed a significant (*F*_(4,20)_ = 235.4, *P* < 0.0001) increase in the SOD levels (0.42 ± 0.0 SOD unit mg^−1^ protein compared with the vincristine control group with 0.074 ± 0.03 SOD unit mg^−1^ protein (Table 2)).

#### 3.8.3. Catalase (CAT) Levels

Administration of vincristine caused a significant decline in catalase levels (*P* < 0.0001) when compared with the naive group. The vincristine control group expressed reduced CAT levels with 0.77 ± 0.18 unit CAT mg^−1^ proteins compared with 7.45 ± 0.29 unit CAT mg^−1^ protein CAT levels of the naïve group. After treatment with pregabalin and BAE, there was a significant and dose-dependent increase (*F*_(4,20)_ = 97.32, *P* < 0.0001, *F*_(420)_ = 168.2, *P* < 0.0001, respectively) in CAT levels (Table 2).

#### 3.8.4. Reduced Glutathione (GSH) Levels

The GSH level in the naïve group was 686.7 ± 89.93 *μ*mol mg^−1^ as against 231.31 ± 16.36 *μ*mol mg^−1^ in the vincristine control group. BAE (1000 mg kg^−1^) treatment caused a significant increase in the GSH levels (*F*_(4,10)_ = 12.14, *P* = 0.0007) with a value of 548.5 ± 19.04 *μ*mol mg^−1^ (Table 2). There was no significant increase in GSH levels in the groups treated with 10 mg kg^−1^ pregabalin and 50 mg kg^−1^ BAE. 100 mg kg^−1^ pregabalin showed a significant (*F*_(4,10)_ = 13.77, *P* = 0.0004) increase in GSH levels with 543.41 ± 44.14 *μ*mol mg^−1^ (Table 2).

#### 3.8.5. Myeloperoxidase Levels

Tissue homogenate from rats in the naïve group showed relatively high MPO levels compared with the homogenate from the vincristine control group. BAE treatment was able to dose-dependently and significantly decrease the MPO levels (*F*_(4,10)_ = 44.21 *P* < 0.0001). Similarly, homogenate from rats treated with pregabalin also showed a significantly (*F*_(4,10)_ = 35.17, *P* < 0.0001) decreased levels of MPO (Table 2).

#### 3.8.6. Malondialdehyde Levels

The vincristine control group exhibited a significant increase in MDA levels compared with the naïve group. Treatment with BAE significantly (*F*_(4,10)_ = 16.41, *P*=0.0002) and dose-dependently decreased the levels of MDA compared with the vincristine control group (Table 2). Similarly, the pregabalin-treated group also showed a significant (*F*_40_ = 12.60, *P*=0.0021) and dose-dependent decrease in the MDA levels compared with the vincristine control group (Table 2).

## 4. Discussion

*Burkea africana* extract (BAE), given orally, elicited a dose-dependent antiallodynic and antihyperalgesic effect in a rat model of peripheral neuropathic pain induced with vincristine. Vincristine is an anticancer agent, which interferes with *β*-tubulin at the vinca domain and alters the cell morphology through inhibition of spindle microtubule formation [27]. Cancer-induced neuropathy (CIN) is characterized by glutamate excitotoxicity due to the rise in sodium and calcium levels and NMDA receptor activation. Also CIN causes downregulation of opioid receptors; hence, opioid analgesics like morphine are insensitive to neuropathic pain [28]. However, pregabalin is efficacious in both experimental, as demonstrated in this study, and clinical settings for alleviating chemotherapy-induced nociception [11, 29–33]. Systemic administration of 0.1 mg kg^−1^ vincristine sulphate excites synthesis and release of cytokines from neuroglial cells leading to inflammation of neurons. The inflammation stimulates the Janus kinase-transcription-3 pathway (Jak-STAT3 pathway) leading to neuropathic pain in rats [27, 33–36]. More so, vincristine treatment alters the nervous system and creates transient transduction by nerve fibers (C- and A*β*-fibers) resulting in a spontaneous pain and unusual sensations both peripherally and centrally [37]. Neuropathic pain is associated with various forms of allodynia including tactile, cold, heat, and mechanical, and this is thought to be mediated through the activation of small diameter fibers (C- and A*δ*-fibers) and large diameter fiber (A*β*-fibers) [38, 39]. Therefore, the extract's inhibitory effects in tactile, cold, and hot as well as mechanical hyperalgesia in this study may be as a result of alteration in the conduction of action potential in the unmyelinated and myelinated C-, A*δ*-, and A*β*-fibers. It could also be that BAE was able to reduce the number of calcium and sodium ions that were actively raised after vincristine injection. Since BAE has already been proven to have anti-inflammatory effect [40], it could have also reduced the inflammation of neurons associated with vincristine-induced neuropathy as well as decreasing NMDA receptor activation. The exact mechanism of action of BAE, however, needs to be established in further studies.

In a rat model of drug-induced peripheral neuropathy, markers of oxidative stress and DNA oxidation increase in systemic circulation, sciatic nerve, and lumbar spinal cord [41]. Oxidative stress is directly involved in the pathogenesis of cancer-induced neuropathic pain [42]. A number of studies have established the co-existence of increased oxidative stress as a central mediator of apoptosis through mitochondria toxicity, neuroinflammation, and biochemical changes [42, 43]. Mitotoxicity evoked by anticancer agents impairs ATP production and cell signaling [44]. This is demonstrated by the innate antioxidant defenses in association with enhanced susceptibility to lipid peroxidation [41–43, 45–48]. More so, studies elsewhere have shown that some anticancer agents (e.g., vincristine) can provoke oxidative stress in chemotherapy [42]. Oxidative stress can influence muscle sensitivity, and reports suggest there may be a reciprocal interaction between oxidative stress and skeletal muscle susceptibility to fatigue and pain [49]. Furthermore, free radicals are known to be associated with pain induction in chronic pain conditions by causing a decrease in the threshold of nociceptors and leading to hyperalgesia [50, 51]. A significant restraint stress-induced decline in the activities of SOD, CAT, and GSH levels follows vincristine treatments [42]. From this study, lipid peroxidation products, malondialdehyde, and protein carbonyl contents were accumulated in the stressed vincristine control animals. Sciatic nerve homogenate (SNH) obtained from this study demonstrated that treatment with BAE inhibited the oxidative stress evidenced in the preservation of the sciatic nerve tissue antioxidant capacity. Endogenous antioxidant markers such as GSH, SOD, and CAT in the SNH were significantly elevated in the BAE-treated animals compared with the vincristine control animals. Saline-treated rats relative to the BAE-treated rats presented with significantly higher levels of MDA, a positive indicator of oxidative stress. This is an indication that potentially damaging processes such as lipid peroxidation and superoxide anion-mediated free radical generation were inhibited by BAE. This apparent antioxidant effect of BAE is consistent with some previous studies reporting in- vitro antioxidant activity of extracts of *B. africana* [52–54]. It can thus be proposed that enhancing the endogenous antioxidant defenses could play part in the effectiveness of BAE in the vincristine-induced neuropathy in rats.

In summary, this study has confirmed that the oral administration of hydroethanolic stem bark extract of *B. africana* exerts antiallodynic and antihyperalgesic effects in vincristine-induced peripheral neuropathy in rats possibly through the modulation of oxidant/antioxidant balance in the sciatic nerve tissue and/or inhibiting the production of inflammatory mediators.

## Figures and Tables

**Table 1:** Phytochemical constituents of the hydroethanolic stem bark extract of *B. africana*.

Secondary metabolite	Inference
Alkaloids	+
Reducing sugars	+
Tannins	+
Flavonoids	+
Saponins	+
Triterpenoids	+
Phytosterols	+

Key: + denotes present.

**Table 2:** Effects BAE and pregabalin on biochemistry parameters of the sciatic nerve and tissue homogenate.

Group	Naïve	Vincristine control	BAE (mg kg^−1^)			Pregabalin (mg kg^−1^)		
			50	500	1000	10	30	100
TPC	2.57 ± 0.27	4.37 ± 0.20	3.12 ± 0.24^*∗*^	3.13 ± 0.18^*∗*^	3.06 ± 0.49^*∗*^	3.41 ± 0.53	2.97 ± 0.35^*∗*^	2.83 ± 0.18^*∗*^
SOD	0.71 ± 0.01	0.07 ± 0.03	0.17 ± 0.05	0.33 ± 0.01^*∗∗∗∗*^	0.43 ± 0.01^*∗∗∗∗*^	0.17 ± 0.01^*∗∗*^	0.37 ± 0.02^*∗∗∗∗*^	0.42 ± 0.00^*∗∗∗∗*^
CAT	7.48 ± 0.29	0.77 ± 0.18	3.62 ± 0.33^*∗∗∗∗*^	4.10 ± 15^*∗∗∗∗*^	4.58 ± 0.21^*∗∗∗∗*^	3.04 ± 0.11^*∗∗∗∗*^	4.06 ± 2.0^*∗∗∗∗*^	4.91 ± 0.10^*∗∗∗∗*^
GSH	686.7 ± 89.93	231.3 ± 16.36	358.1 ± 55.02	507.40 ± 31.97^*∗*^	548.5 ± 19.04^*∗∗*^	332.80 ± 18.70	492.10 ± 30.78^*∗*^	543.40 ± 44.14^*∗∗*^
MDA	30.21 ± 6.08	335.60 ± 46.35	228.50 ± 23.77	163.10 ± 10.96^*∗∗∗*^	98.06 ± 36.95^*∗∗∗∗*^	177.20 ± 47.45^*∗∗∗*^	144.0 ± 24.64^*∗∗∗∗*^	99.20 ± 34.36^*∗∗∗∗*^
MPO	62.48 ± 10.02	230.50 ± 16.27	195.80 ± 6.78	133.30 ± 4.99^*∗∗*^	106.70 ± 9.02^*∗∗∗*^	142.90 ± 10.10^*∗*^	120.90 ± 5.17^*∗*^	107.80 ± 7.20^*∗∗*^

Biochemical parameters were estimated from the sciatic nerve and accompanying tissue homogenate. ^*∗∗∗∗*^*P* < 0.0001; ^*∗∗∗*^*P* < 0.001; ^*∗∗*^*P* < 0.01; ^*∗*^*P* < 0.05 compared with the control group (ordinary 1-way ANOVA complemented with Dunnett's post hoc test). Each data presented are mean ± S.E.M (*n* = 5). TPC = total protein content, SOD = super oxide dismutase, CAT = catalase, GSH = reduced glutathione, MDA = malondialdehyde, and MPO = myeloperoxide.

## Data Availability

The data used to support the findings of this study are available from the corresponding author upon request.
